# The Late Mr. Reginald Lund

**Published:** 1907-11-23

**Authors:** 

**Affiliations:** Chairman Charing Cross Hospital.


### THE LATE MR. REGINALD LUND.

Sir,—Among the numerous readers of your journal there are many in charitable, professional, and artistic circles who are well acquainted with the valuable work performed by the late Mr. Reginald Gregory Lund as Organising Secretary of the League of Mercy, and at one time Secretary of the Special Appeal Committee of Charing Cross Hospital.

I shall be greatly obliged, therefore, if you will permit me, through the medium of your columns, to make it well known that a meeting of intimate friends and admirers of the late Mr. Lund was held here on the 12th inst., at which it was unanimously decided to raise a fund to his memory, to be called "The Reginald Lund Memorial Fund," the proceeds to be devoted to the benefit of his widow and children.

Donations will be gratefully received either by Dora, Countess of Chesterfield, 26 Egerton Gardens, S.W., or by

Yours faithfully,

Kilmorey,

Chairman Charing Cross Hospital.

